# Sigma‐1 receptor protects against ferroptosis in hepatocellular carcinoma cells

**DOI:** 10.1111/jcmm.14594

**Published:** 2019-09-10

**Authors:** Tao Bai, Pengxu Lei, Hao Zhou, Ruopeng Liang, Rongtao Zhu, Weijie Wang, Lin Zhou, Yuling Sun

**Affiliations:** ^1^ Department of Hepatobiliary and Pancreatic Surgery, School of Medicine, The First Affiliated Hospital of Zhengzhou University, Institute of Hepatobiliary and Pancreatic Diseases Zhengzhou University Zhengzhou China; ^2^ Department of Gastroenterology The First Affiliated Hospital of Zhengzhou University Zhengzhou China

**Keywords:** ferroptosis, hepatocellular carcinoma, sigma‐1 receptor, sorafenib

## Abstract

Sigma‐1 receptor (S1R) regulates reactive oxygen species (ROS) accumulation via nuclear factor erythroid 2‐related factor 2 (NRF2), which plays a vital role in ferroptosis. Sorafenib is a strong inducer of ferroptosis but not of apoptosis. However, the mechanism of sorafenib‐induced ferroptosis in hepatocellular carcinoma (HCC) remains unclear. In this study, we found for the first time that sorafenib induced most of S1Rs away from nucleus compared to control groups in Huh‐7 cells, and ferrostatin‐1 completely blocked the translocation. S1R protein expression, but not mRNA expression, in HCC cells was significantly up‐regulated by sorafenib. Knockdown of NRF2, but not of p53 or hypoxia‐inducible factor 1‐alpha (HIF1α), markedly induced S1R mRNA expression in HCC cells. Inhibition of S1R (by RNAi or antagonists) increased sorafenib‐induced HCC cell death in vitro and in vivo. Knockdown of S1R blocked the expression of glutathione peroxidase 4 (GPX4), one of the core targets of ferroptosis, in vitro and in vivo. Iron metabolism and lipid peroxidation increased in the S1R knockdown groups treated with sorafenib compared to the control counterpart. Ferritin heavy chain 1 (FTH1) and transferrin receotor protein 1 (TFR1), both of which are critical for iron metabolism, were markedly up‐regulated in HCC cells treated with erastin and sorafenib, whereas knockdown of S1R inhibited these increases. In conclusion, we demonstrate that S1R protects HCC cells against sorafenib and subsequent ferroptosis. A better understanding of the role of S1R in ferroptosis may provide novel insight into this biological process.

## 1 INTRODUCTION

Hepatocellular carcinoma (HCC) is a common type of cancer that is ranked sixth in prevalence and third in mortality worldwide.^1^ Because of the complexity of the disease and many newly discovered treatments, systemic therapy plays an increasingly important role in HCC treatment. Neither surgical nor non‐surgical treatment yields satisfactory results in advanced HCC patients. Several targeted agents have been developed, but only sorafenib and regorafenib have been proven to successfully prolong the survival of HCC patients.^2^, ^3^, ^4^ Sorafenib was the first approved systemic therapy for advanced HCC patients based on the positive results of two randomized trials,^2^, ^3^ and subsequent cohort studies confirmed the efficacy in clinical practice.^5^, ^6^

Recently, it was reported that sorafenib is a strong inducer of ferroptosis but not of apoptosis.^7^, ^8^ Ferroptosis is a newly discovered cell death that differs from apoptosis, necroptosis and autophagy morphologically, genetically and biochemically.^9^, ^10^ It seems that ferroptosis occurs in an iron‐dependent accumulation of reactive oxygen species (ROS) way.^9^, ^11^, ^12^ Glutathione (GSH) and the downstream protein glutathione peroxidase 4 (GPX4) were proved to regulate the metabolism of iron, lipid peroxidation and subsequent ferroptosis.^13^ It was also reported that the p62‐Keap1‐NRF2 pathway regulates the susceptibility of HCC cells to ferroptosis by regulating the expression of NRF2.^7^ However, the regulation networks of ferroptosis remain mostly unknown.

The sigma receptors are non‐opioid proteins. In addition to central nervous system, S1Rs are also found in the liver, pancreas and cancer cells.^14^ Although it has been suggested that S1Rs take part in many cellular processes, the function and regulation of S1Rs in the liver and cancer cells remain elusive. Recent studies reported that S1Rs suppress the production of ROS in many organs, possibly by activating antioxidant response elements and decreasing oxidized GSH and glutamate.^15^, ^16^ A previous study also provided credible evidence that S1Rs regulate ROS by modulating the NRF2‐Keap1 pathway and system X_c_^−^,^14^ both of which are critical in ferroptosis.

Although accumulating evidence suggests that S1Rs may be involved in ferroptosis,^14^, ^16^, ^17^, ^18^ the exact role and function of S1Rs in ferroptosis remain unclear. In this study, we confirmed that through modulating GPX4, iron metabolism and ROS accumulation, inhibition of S1R strengthened the anticancer effect of sorafenib in HCC cells in vitro and in vivo. In general, our work identified a novel and direct link between S1R and ferroptosis.

## 2 MATERIALS AND METHODS

### 2.1 Reagents

Antibodies to NRF2 (ab62352) and GPX4 (ab125066), as well as necrosulfonamide (ab143839), were obtained from Abcam (Shanghai, China). Antibody to S1R (sc137075) was obtained from Santa Cruz (Shanghai, China). Erastin (No. S7242), sorafenib (No. S7397), ZVAD‐FMK (No. S7023) and ferrostatin‐1 (No. S7243) were obtained from Selleck (Shanghai, China). All‐trans retinoic acid (ATRA; R2625), trigonelline (T5509), PRE‐084 (P2607), BD1063 (SML0276) and BD1047 (B8562) were obtained from Sigma‐Aldrich (Shanghai, China).

### 2.2 Cell culture

Hep G2, Huh‐7, SMMC‐7721 and PLC/PRF/5 cells were purchased from the Type Culture Collection of the Chinese Academy of Sciences (Shanghai, China). These cells were cultured in Dulbecco's modified Eagle's medium (Hep G2, Huh‐7 and PLC/PRF/5) or RPMI 1640 medium (SMMC‐7721) supplemented with 10% foetal bovine serum (HyClone) and 100 U/mL penicillin and streptomycin in a humidified incubator with 5% CO_2_ and 95% air.

### 2.3 Cell viability assay

Cell viability was evaluated using a Cell Counting Kit‐8 (CCK‐8) (Dojindo Laboratories, Shanghai, China) according to the manufacturer's instructions. WST‐8 [2‐(2‐methoxy‐4‐nitrophenyl)‐3‐(4‐nitrophenyl)‐5(2,4‐disulfophenyl)‐2H‐tetrazolium monosodium salt] is a sensitive next‐generation reagent that serves as an indicator of NADH. Under certain conditions, NADH reduces WST‐8 to produce a water‐soluble formazan dye, which is used as a cell viability indicator in cell proliferation and death assays by measuring the absorption at 450 nm.

### 2.4 Clonogenic survival assay

A colony formation assay was performed to monitor long‐term cell survival. Cells were seeded at 500 cells/well in 24‐well plates and treated with individual chemotherapeutic drugs for 24 hours. The medium was changed every 3 days. After 2 weeks, colonies were visualized by crystal violet staining after fixation with 4% paraformaldehyde as previously described.^19^

### 2.5 Immunofluorescence (IF)

Cells were seeded in 24‐well plates and treated with individual chemotherapeutic drugs for 24 hours. Then, the cells were fixed with 4% paraformaldehyde for 15 minutes and permeabilized with 0.1% Triton X‐100 for 30 minutes. After incubation overnight with the S1R antibody (control groups without S1R antibody), the cells were washed with PBS three times. Then, the cells were incubated with the secondary antibody for 1 hour. Finally, DAPI was added to stain the cell nucleus, and the cells were detected by luorescence microscope (400×, Olympus).

### 2.6 Western blot analysis

Cells were lysed with radioimmunoprecipitation assay (RIPA) buffer to harvest protein. Total protein from each sample was separated by 8%‐12% SDS‐polyacrylamide gel electrophoresis and then transferred to polyvinylidene fluoride membranes (pore size 0.45 μm), which were subsequently incubated with the appropriate primary antibody. After incubation with the secondary antibody, the blots were visualized using an enhanced chemiluminescence system.

### 2.7 RNAi and gene transfection

Human NRF2‐shRNA (gatccccCCGGCATTTCACTAAACACAACTCGAGTTGTGTTTAGTGAAATGCCGGttttt), human p53‐shRNA (gatccccCGGCGCACAGAGGAAGAGAATCTCGAGATTCTCTTCCTCTGTGCGCCGttttt), human HIF1α‐shRNA (gatccccGTGATGAAAGAATTACCGAATCTCGAGATTCGGTAATTCTTTCATCACttttt), human S1R‐shRNA_1 (gatccccGATACCATCATCTCTGGCATGCCAGAGATGATGGTATCttttt), human S1R‐shRNA_2 (gatccccCACATGGATGGTGGAGTACGTACTCCACCATCCATGTGttttt) and control shRNA were obtained from GenePharma. Transfections were performed with Lipofectamine™ 3000 (L3000‐008, Invitrogen) according to the manufacturer's instructions.

### 2.8 Quantitative real‐time polymerase chain reaction

Total RNA isolation and quantitative RT‐PCR (Q‐PCR) amplification were performed as previously described.^20^ Briefly, first‐strand cDNA synthesis was performed by using a Reverse Transcription System Kit according to the manufacturer's instructions (RR820A, Takara Biomedical Technology). cDNA was amplified with specific primers (human S1R: 5′‐AGTATGTGCTGCTCTTCGGC‐3′ and 5′‐CTCCACCATCCATGTGTTTG‐3′; human p53: 5′‐ACCACCATCCACTACAACTACAT‐3′ and 5′‐CAGGACAGGCACAAACACG‐3′; human HIF‐1α: 5′‐AGTGTACCCTAACTAGCCGA‐3′ and 5′‐CACAAATCAGCACCAAGC‐3′; human NRF2: 5′‐GTCAGCGACGGAAAGAGTA‐3′ and 5′‐ACCTGGGAGTAGTTGGCA‐3′; human divalent metal transporter 1 (DMT1): 5′‐TTCTTATGAGCATTGCCTAC‐3′ and 5′‐GACCTTGGGATACTGACG‐3′; human FTH1: 5′‐CGCCAGAACTACCACCAG‐3′ and 5′‐TTCAAAGCCACATCATCG‐3′; human GPX4: 5′‐GAAGCAGGAGCCAGGGAGT‐3′ and 5′‐ACGCAGCCGTTCTTGTCG‐3′; human HO‐1:5′‐TTTGAGGAGTTGCAGGAGC‐3′ and 5′‐AGGACCCATCGGAGAAGC‐3′; and human TFR1: 5′‐GCTTTCCCTTTCCTTGCA‐3′ and 5′‐CGAACTGACCAGCGACCT‐3′).

### 2.9 Iron assay

The intracellular iron concentration was assessed using an iron colorimetric assay kit purchased from Biovision according to the manufacturer's instructions.

### 2.10 Lipid peroxidation assay

The intracellular malondialdehyde (MDA) concentration was assessed using a lipid peroxidation colorimetric assay kit purchased from Biovision according to the manufacturer's instructions.

### 2.11 Glutathione assay

The intracellular glutathione (GSH) level was assessed using a GSH colorimetric assay kit purchased from Biovision according to the manufacturer's instructions.

### 2.12 Animal models

All animal experiments were approved by the Institutional Review Board of the First Affiliated Hospital of Zhengzhou University and performed in accordance with the National Institutes of Health guide for the care and use of Laboratory animals.

To generate murine subcutaneous tumours, 1 × 10^7^ control shRNA or S1R‐knockdown Huh‐7 cells in 200 μL of PBS were injected subcutaneously to the right of the dorsal midline. At day seven, the mice were randomly divided into groups and treated with sorafenib (10 mg/kg/intraperitoneal injection (i.p.), once every other day) for 2 weeks. On day 28, tumours were removed. Tumours were measured every 3 days, and tumour volume was calculated using the formula length × width^2^ × π/6.

### 2.13 Statistical analysis

All data are expressed as the mean ± SD of three independent experiments. Data were analysed using unpaired Student's *t* tests for comparisons of two groups or ANOVA LSD tests for comparisons among multiple groups. Significance was defined as *P* < .05.

## 3 RESULTS

### 3.1 Sorafenib induces S1R protein expression in human HCC cells

**Figure 1:**
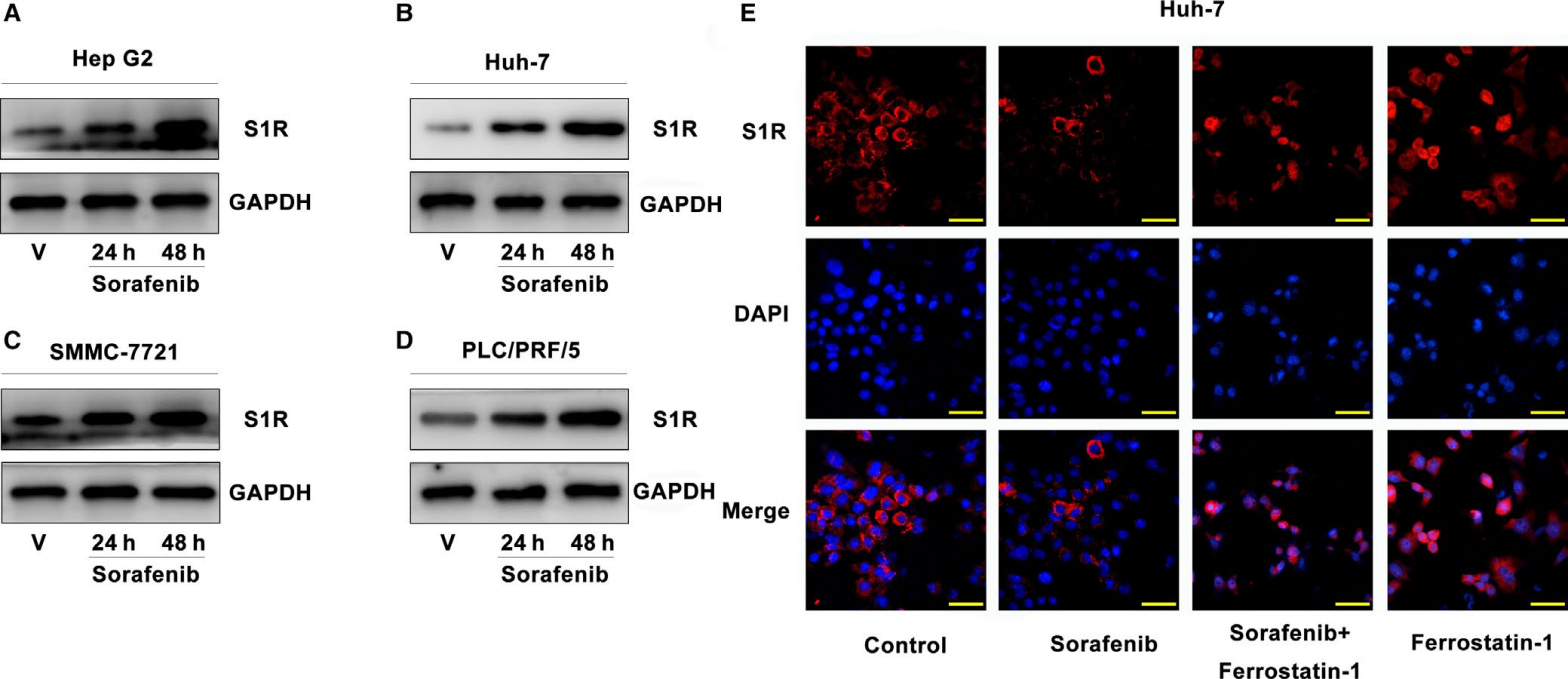
Sorafenib induces S1R protein expression in human HCC cells. A‐D, HCC cells were treated with sorafenib (5 μmol/L) for 24‐48 h, and the protein expression of S1R was assayed using Western blot analysis. E, Huh‐7 cells were treated with or without sorafenib and ferrostatin‐1, and immunofluorescence staining was performed to exhibit the locations of S1Rs (Bars = 50 μm)

Western blot analysis revealed that S1R protein levels were significantly increased in SMMC‐7721 and PLC/PRF/5 cells following treatment with sorafenib (Figure 1C,D), as they were in Hep G2 and Huh‐7 cells (Figure 1A,B).^21^ Furthermore, sorafenib up‐regulated S1R protein levels in these HCC cells in a time‐dependent manner (Figure 1A‐D). Then, Huh‐7 cells were treated with or without sorafenib and ferrostatin‐1 (a ferroptosis inhibitor), and immunofluorescence staining was performed. We observed interesting results that sorafenib induced most of S1Rs away from nucleus compared to control groups in Huh‐7 cells, and ferrostatin‐1 completely blocked the translocation. (Figure 1E). These data indicate that sorafenib induces S1R expression in a time‐dependent manner in human HCC cells, and translocation of S1Rs in Huh‐7 cells.

### 3.2 Inhibition of NRF2 leads to increased S1R expression

**Figure 2:**
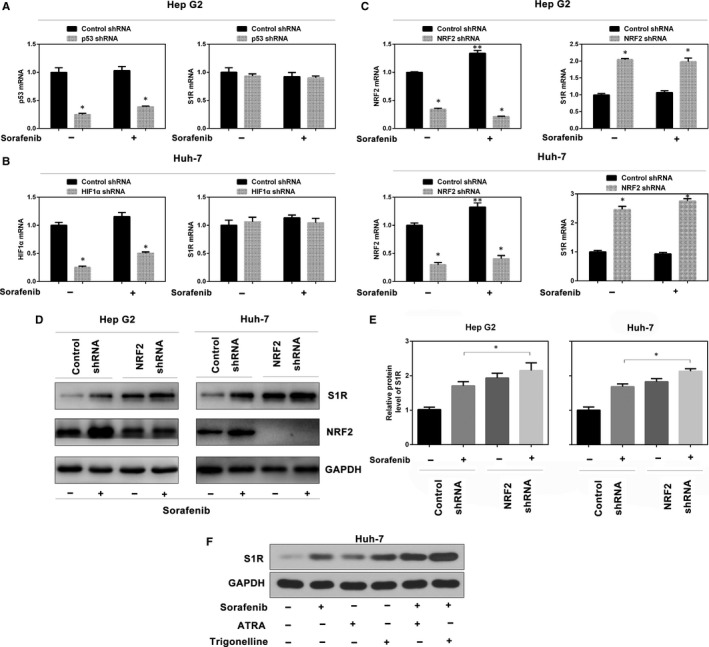
Inhibition of NRF2 leads to increased S1R expression. A‐C, Indicated HCC cells were treated with sorafenib (5 μmol/L) for 24 h, and the mRNA levels of indicated genes were assayed by Q‐PCR (n = 3, *P < .05 vs control shRNA group, **P < .05 vs no‐drug control shRNA group). D, Knockdown of NRF2 by shRNA augmented sorafenib‐induced S1R protein expression by Western blot analysis. E, In parallel, the relative intensity of the Western blot band of S1R was quantified using ImageJ densitometry software (n = 3, *, *P* < .05). F, Huh‐7 cells were treated with sorafenib (5 μmol/L) with or without all‐trans retinoic acid (ATRA, 1 μmol/L) and trigonelline (0.5 μmol/L) for 24 h, and S1R protein expression was assayed using Western blot analysis

Sorafenib regulates the activity of the transcription factors p53,^22^ hypoxia‐inducible factor 1‐alpha (HIF1α)^23^ and NRF2.^7^ To determine which transcription factor regulates S1R expression, target‐specific shRNAs against p53, HIF1α and NRF2 were transfected into HCC cells. Interestingly, except for NRF2, the mRNA levels of p53, HIF1α and S1R were all not significantly affected by sorafenib (Figure 2A‐C). Combining the data from above (Figures 1A‐D and 2A‐C), the results suggest a post‐transcriptional mechanism for S1R to regulate ferroptosis. However, knockdown of NRF2, but not of p53 or HIF1α, significantly induced S1R mRNA expression (Figure 2A‐C). Furthermore, knockdown of NRF2 also augmented sorafenib‐induced S1R protein expression in Hep G2 and Huh‐7 cells (Figure 2D,E). In addition, two NRF2 inhibitors (ATRA^24^ and trigonelline^25^) also enhanced sorafenib‐induced S1R protein expression in Huh‐7 cells (Figure 2F). Thus, these data suggest that inhibition of NRF2 accelerates sorafenib‐induced S1R protein expression in HCC cells.

### 3.3 Suppression of S1R expression increases the sorafenib sensitivity of HCC cells

**Figure 3:**
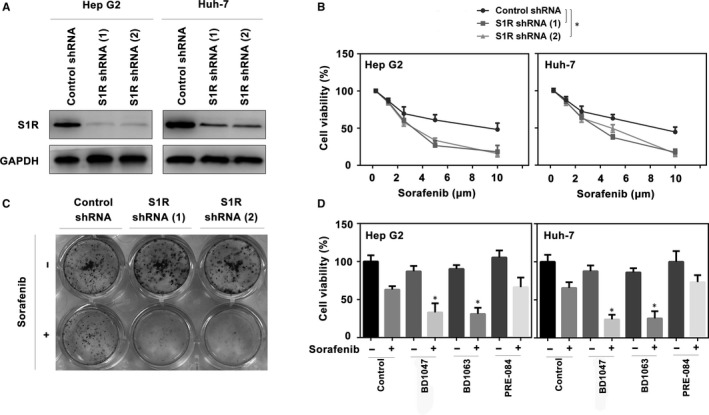
Suppression of S1R expression increases the sorafenib sensitivity of HCC cells. A, Western blot analysis of S1R expression in indicated S1R knockdown HCC cells. B, Indicated S1R knockdown HCC cells were treated with sorafenib (1.25‐10 μmol/L) for 24 h and cell viabilities were assayed (n = 3, *P < .05). C, Clonogenic survival assay. Indicated Huh‐7 cells were treated with sorafenib (5 μmol/L) for 24 h, and then 500 cells were plated into 24‐well plates. Colonies were visualized by crystal violet staining 2 wk later. D, Indicated HCC cells were treated with sorafenib (5 μmol/L), with or without BD1063 (10 μmol/L), BD1047 (10 μmol/L) or PRE‐084 (20 μmol/L) for 24 h and cell viability was assayed (n = 3, *P < .05 vs control group)

To explore whether S1R expression influences the activity of sorafenib in HCC cells, two different shRNAs targeting S1R were transfected into Hep G2 and Huh‐7 cells (Figure 3A). RNAi‐mediated suppression of S1R expression significantly increased sorafenib‐induced cell death, as demonstrated by cell viability assays (Figure 3B). A colony formation assay also indicated that the suppression of S1R significantly strengthened the anticancer activity of sorafenib in HCC cells (Figure 3C). Similar to our previous study,^21^ another two S1R antagonists (BD1063 and BD1047)^26^ increased sorafenib‐induced HCC cell death, while the S1R agonist PRE‐084 had no effect on cell death (Figure 3D). In general, these findings demonstrate that S1R inhibition increases the sensitivity of HCC cells to sorafenib.

### 3.4 S1R protects against ferroptosis in HCC cells

**Figure 4:**
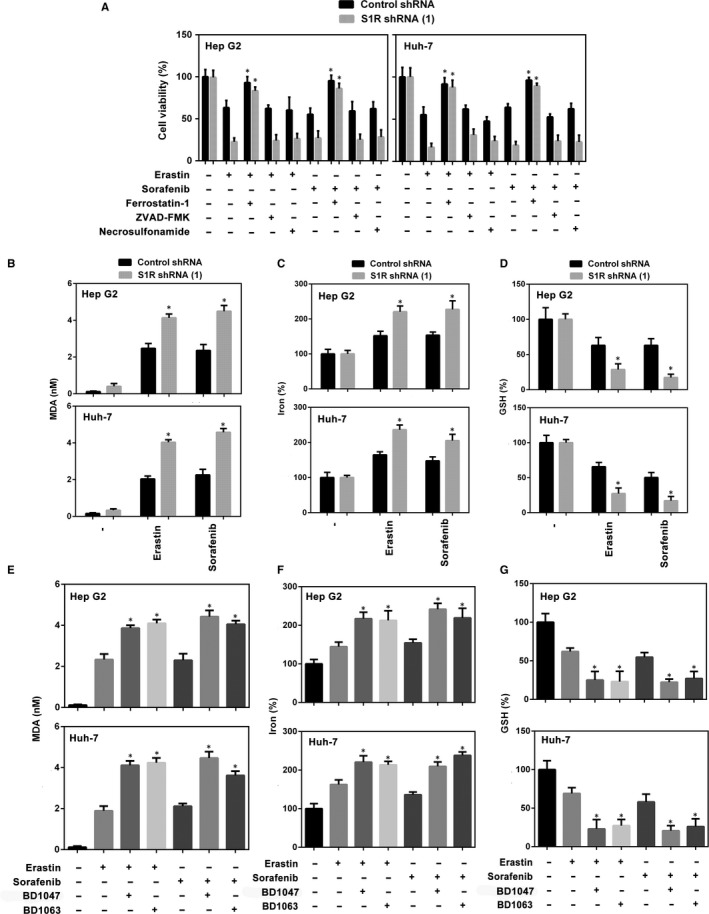
S1R protects against ferroptosis in HCC cells. A, Indicated HCC cells were treated with erastin (10 μmol/L) and sorafenib (5 μmol/L) with or without cell death inhibitors (ferrostatin‐1, 1 μmol/L; ZVAD‐FMK, 10 μmol/L; necrosulfonamide, 0.5 μmol/L) for 24 h and cell viability was assayed (n = 3, *P < .05 vs erastin or sorafenib treatment group). B‐D, Indicated HCC cells were treated with erastin (10 μmol/L) or sorafenib (5 μmol/L) for 24 h. The levels of malondialdehyde (MDA), Fe2+ and glutathione (GSH) were assayed (n = 3, *P < .05 vs control shRNA group). E‐G, Indicated HCC cells were treated with erastin (10 μmol/L) or sorafenib (5 μmol/L) with or without BD1063 (10 μmol/L), BD1047 (10 μmol/L) or PRE‐084 (20 μmol/L) for 24 h. The levels of MDA, Fe2+ and GSH were assayed (n = 3, *P < .05 vs erastin or sorafenib treatment group)

We explored the mechanism by which S1R mediates ferroptosis. S1R‐knockdown HCC cells were treated with several cell death inhibitors, and the results showed that the ferroptosis inhibitor (ferrostatin‐1^9^) significantly blocked sorafenib‐induced cell death in both control and S1R‐knockdown cells. However, ZVAD‐FMK (apoptosis inhibitor) and necrosulfonamide (necroptosis inhibitor) did not show a significant effect in the same experiment (Figure 4A). In addition, knockdown of S1R augmented cell death induced by erastin (classical ferroptotic inducer), which was blocked by ferrostatin‐1 but not by ZVAD‐FMK or necrosulfonamide (Figure 4A). These data suggest that S1R protects against ferroptosis in HCC cells.

Then, we explored the role of S1R in regulating lipid peroxidation and iron metabolism. As expected, the level of MDA (a typical lipid peroxidation product) was significantly increased in S1R‐knockdown cells treated with erastin and sorafenib compared with the control counterpart (Figure 4B). Iron takes part in the Fenton reaction and produces ROS to induce ferroptosis.^27^ In this study, knockdown of S1R significantly increased Fe^2+^ levels in HCC cells treated with erastin and sorafenib compared with the control counterpart (Figure 4C). Similar to our previous study,^21^ another two S1R antagonists (BD1063 and BD1047) increased erastin‐ and sorafenib‐induced MDA production and Fe^2+^ levels (Figure 4E,F). These findings suggest that S1R protects against ferroptosis in HCC cells by modulating lipid peroxidation and iron metabolism.

In addition to ROS and iron, GSH also plays a key role in ferroptosis by mediating lipid peroxidation.^12^, ^14^, ^28^ We found that knockdown of S1R significantly enhanced intracellular GSH consumption in HCC cells treated with sorafenib or erastin compared with the control counterpart (Figure 4D). As expected, S1R antagonists (BD1063 and BD1047) also enhanced intracellular GSH consumption in HCC cells treated with sorafenib or erastin (Figure 4G). Thus, these data suggest that S1R protects against ferroptosis in HCC cells by inhibiting GSH consumption‐mediated lipid peroxidation.

### 3.5 S1R influences many downstream targets in ferroptosis

**Figure 5:**
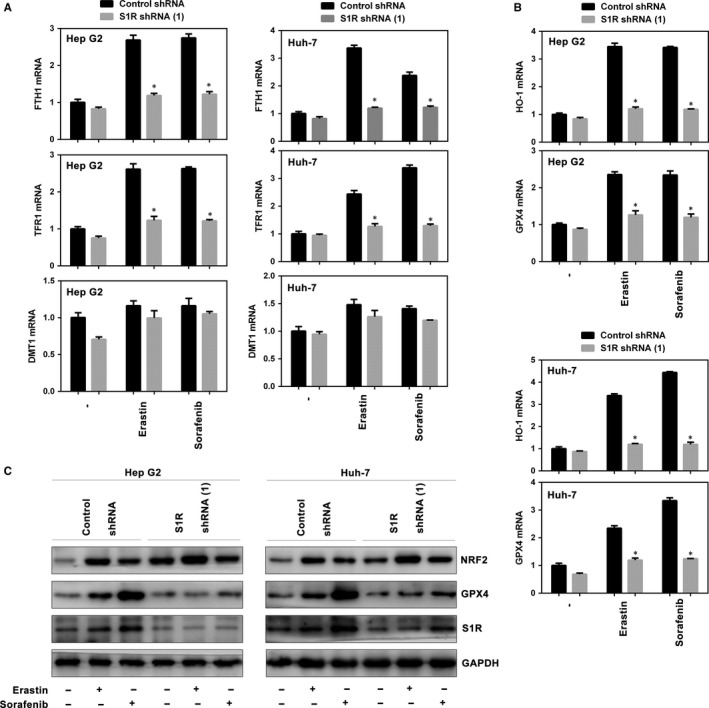
S1R influences many downstream targets in ferroptosis. A‐B, Indicated HCC cells were treated with erastin (10 μmol/L) or sorafenib (5 μmol/L) for 24 h. The mRNA levels of indicated genes were assayed (n = 3, *P < .05 vs control shRNA group). C, Indicated HCC cells were treated with erastin (10 μmol/L) or sorafenib (5 μmol/L) for 24 h and the protein expression of indicated genes were assayed using Western blot analysis

Ferroptosis is a novel form of programmed cell death that has a close relationship with iron metabolism.^12^, ^27^, ^29^, ^30^ We explored the role of S1R in signalling pathways related to ferroptosis, especially in the iron metabolism pathway. Among the iron metabolism genes (FTH1, TFR1 and DMT1), ferritin heavy chain 1 (FTH1) and transferrin receotor protein 1 (TFR1) were markedly up‐regulated in HCC cells treated with erastin and sorafenib, whereas knockdown of S1R inhibited these increases (Figure 5A). Furthermore, knockdown of S1R inhibited the mRNA levels of haeme oxygenase 1 (HO‐1) and GPX4, important targets in ferroptosis,^7^, ^13^, ^31^, ^32^ in HCC cells compared with the control counterpart (Figure 5B). Then, we investigated whether NRF2 and GPX4 protein levels are altered in S1R‐knockdown cells treated with erastin or sorafenib. GPX4 levels were blocked compared with the control counterpart as expected, while NRF2 levels were not markedly affected (Figure 5C). Collectively, these findings indicate that S1R influences key targets in ferroptosis.

### 3.6 Targeting S1R strengthens the anticancer activity of sorafenib in vivo

**Figure 6:**
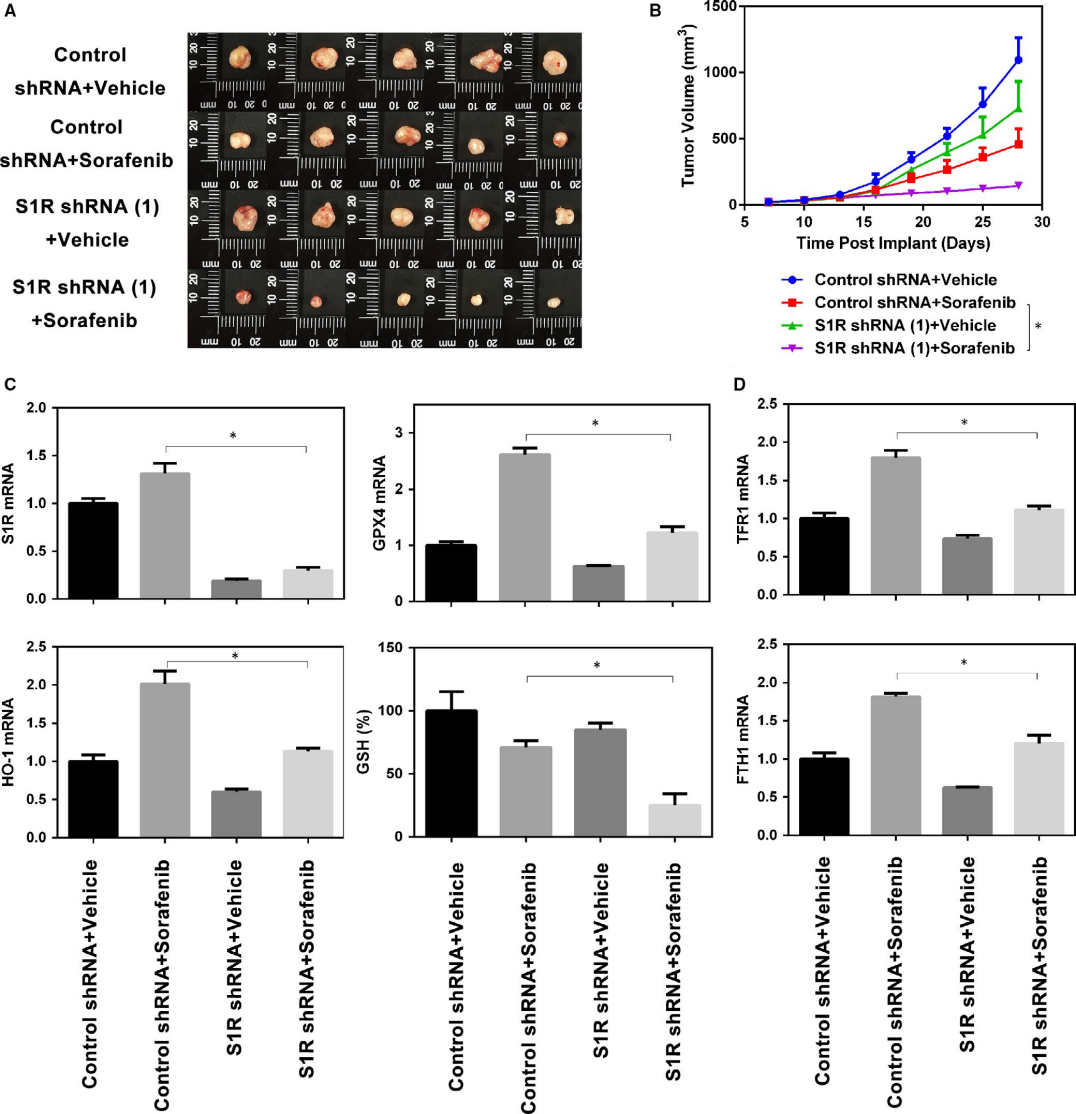
Targeting S1R strengthens the anticancer activity of sorafenib in vivo. A, B, Nude mice were injected subcutaneously with indicated Huh‐7 cells (1 × 10^7^ cells/mouse) and treated with sorafenib (10 mg/kg/i.p., once every other day) and vehicle at day seven for 2 weeks (n = 5 mice/group). Tumour volume was calculated every 3 d. C, D, In parallel, the levels of GSH and indicated genes mRNA in isolated tumours at day 28 were assayed (*P < .05)

To explore whether the inhibition of S1R expression strengthens the anticancer activity of sorafenib in vivo, S1R‐knockdown Huh‐7 cells were implanted subcutaneously in the right flank of nude mice. Beginning on day seven, the mice were treated with sorafenib once every other day. Sorafenib effectively reduced the size of tumours formed by S1R‐knockdown cells compared with those formed by control cells (Figure 6A,B). Then, we investigated the mRNA levels of several targets (S1R, HO‐1, GPX4, FTH1 and TFR1) and GSH levels in isolated tumours; the expression and levels of these molecules were effectively blocked in S1R‐knockdown tumours compared with the control counterpart (Figure 6C,D). These findings indicate that, in HCC, S1R knockdown increases GSH consumption and ferroptosis in vivo, which manifests as increased anticancer activity of sorafenib.

## 4 DISCUSSION

In the treatment of various types of cancer, many studies have focused on seeking novel targeted therapies, while this study explored some mechanisms of ferroptosis occurring in HCC cells exposed to sorafenib. In this study, we provide novel evidence that S1R is a negative regulator of ferroptosis in human HCC cells, which modulates many targets involved in ROS and iron metabolism, as well as the most critical ferroptotic target, GPX4. Inhibition of S1R markedly accelerates erastin‐ and sorafenib‐induced lipid production and subsequent ferroptosis in HCC cells in vitro and in vivo.

Previous studies on S1R focused on the central nervous system.^14^, ^26^, ^33^, ^34^ In cancer cells, S1R protein expression is induced by sorafenib, in turn protecting cells from accumulation of ROS and subsequent ferroptosis. Therefore, S1R may play a dual role in HCC. On the one hand, S1R maintains a stable environment of free radicals and oxidative stress, which obviously prevents the initiation of HCC. On the other hand, S1R can be up‐regulated by sorafenib to protect cancer cells from ferroptosis.

Recent studies have reported that S1R regulates ROS via NRF2, and S1R seems to play a role of cytoprotection in normal cells.^14^ NRF2 is a critical regulator of antioxidant response. It has been demonstrated that the p62‐Keap1‐NRF2 pathway negatively regulates ROS and iron metabolism to protect against ferroptosis in HCC cells.^7^ Therefore, S1R functions similarly to NRF2. Furthermore, in our work, NRF2 in turn negatively modulates S1R gene expression (Figure 2C‐E). In HCC cells exposed to sorafenib, due to NRF2 inactivation and subsequent accumulation of ROS, S1R expression is up‐regulated to protect HCC cells from ferroptosis. In addition, ferroptosis inducers (eg erastin and sorafenib) markedly up‐regulated S1R protein expression, but not mRNA expression, suggesting that S1R plays a transcription‐independent role in ferroptosis. Interestingly, our experiments drew a different result from another team's work^7^ that there was a significant increase in NRF2 mRNA levels on sorafenib treatment compared with the control group (Figure 2C). Together with the above findings, suggesting that NRF2 and S1R have similar patterns in maintaining the redox balance, both proteins are up‐regulated in HCC cells treated with sorafenib, and either inhibition of NRF2 or S1R accelerates ferroptosis, in which they regulate each other.

It was known that sorafenib‐induced ferroptosis is independent of the status of oncogenes.^35^ Several targets regulate ferroptosis through modulating ROS accumulation and iron metabolism. Stable intracellular concentrations of GSH protect cells against oxidative stress responses and ferroptosis. Erastin inactivates GPX4 through depleting GSH. GPX4 promotes the reduction of lipid peroxides in cells on condition of ferroptosis.^13^, ^32^ Obviously, GPX4 protects cells from oxidative stress, and knockdown of GPX4 indeed induces ROS accumulation and subsequent ferroptosis.^13^ Unlike erastin and sorafenib, RSL3, a class 2 ferroptosis inducer, directly binds and inactivates GPX4 without influencing GSH levels.^9^, ^36^ Therefore, many studies consider GPX4 to be the principal target in ferroptosis regardless of the upstream genes.^11^, ^13^, ^31^ We found GPX4 expression is inhibited in S1R‐knockdown groups both in vitro and in vivo, strongly suggesting that S1R may be at upstream to GPX4 in ferroptotic regulatory networks. It remains unknown whether any intermediate target exists between S1R and GPX4 in the pathway.

Iron overload directly cause ROS accumulation and ferroptosis, which was the main distinction from other programmed cell deaths^9^, ^11^, ^12^ (eg apoptosis and necroptosis). Excessive iron generated by the Fenton reaction is the last step to generate ROS accumulation. Inhibition of S1R significantly blocked the increases of FTH1 and TFR1 induced by erastin and sorafenib (Figure 5A), strongly indicating that S1R prevents ROS accumulation by negatively regulating iron metabolism. In addition to causing ROS accumulation, iron overload in liver is also a carcinogenic factor by modulating the immune system.^37^ So, iron also may play a dual role in the liver in oncogenesis and cancer cell death, like S1R.

Another interesting finding was the translocation of S1Rs in Huh‐7 cells in treatment with sorafenib or/with ferrostatin‐1. We provide the first evidence that sorafenib induces S1Rs translocation, and ferrostatin‐1 completely blocked it. We know that ferrostatin‐1 could also block ferroptosis entirely. What remains unknown is the role of S1Rs translocation in antioxidation and inhibiting ferroptosis.

In summary, we demonstrated for the first time that S1R protects HCC cells against sorafenib and subsequent ferroptosis. Inhibition of S1R by RNAi and antagonists markedly increased the anticancer activity of sorafenib by modulating the expression of GPX4, iron metabolism and ROS. Thus, a better understanding of the role of S1R may provide novel insight into ferroptosis. Future work is needed to determine if there exists any intermediate target between S1R and GPX4 in the regulatory networks.

## CONFLICT OF INTEREST

The authors declare no conflicts of interest.

## AUTHOR CONTRIBUTIONS

YS and TB designed the experiments. TB, PL, HZ and RL performed the experiments. RZ and WW analysed the data. TB wrote the draft. YS and LZ checked and revised the manuscript. In the end, we would like to thank Dr Shusen Zheng for his support and help in our work.

## ETHICAL APPROVAL

All animal experiments were approved by the Institutional Review Board of the First Affiliated Hospital of Zhengzhou University and performed in accordance with the National Institutes of Health guide for the care and use of Laboratory animals.

## Data Availability

The data that support the findings of this study are available from the corresponding author upon reasonable request.
